# The Devil We Don't Know: Investigating Habitat and Abundance of Endangered Giant Devil Rays in the North-Western Mediterranean Sea

**DOI:** 10.1371/journal.pone.0141189

**Published:** 2015-11-18

**Authors:** Giuseppe Notarbartolo di Sciara, Giancarlo Lauriano, Nino Pierantonio, Ana Cañadas, Greg Donovan, Simone Panigada

**Affiliations:** 1 Tethys Research Institute, Viale G.B. Gadio 2, 20121, Milano, Italy; 2 Istituto Superiore per la Protezione e la Ricerca Ambientale, Via Vitaliano Brancati 48, 00144, Roma, Italy; 3 ALNILAM Research and Conservation Ltd, Cándamo 116, 28240 Hoyo de Manzanares, Madrid, Spain; 4 The International Whaling Commission, The Red House, 135 Station Road, Impington, Cambridge, Cambridgeshire, CB24 9NP, United Kingdom; Aristotle University of Thessaloniki, GREECE

## Abstract

The giant devil ray *Mobula mobular*, the only Mediterranean mobulid, is subject to mortality caused by directed and accidental captures in fisheries throughout the region. Whilst the combination of human impacts, limited range and a low reproductive potential is not inconsistent with its endangered listing, there are insufficient data to enable a quantitative assessment of trends. Without this, it is difficult to assess and prioritise threats and develop effective conservation actions. Using results from aerial surveys conducted between 2009 and 2014 over the Ligurian, Corsican, Sardinian, northern and central Tyrrhenian seas (626,228 km^2^), this study provides the first quantitative information on giant devil ray abundance and habitat choice in the western Mediterranean. Devil rays were observed in all seasons except winter, with their estimated abundance in the study area peaking in summer. The overall uncorrected mean density in the study area during summer was estimated at 0.0257 individuals km^-2^ (range: 0.017–0.044), resulting in a total abundance estimate of 6,092 (12.7%CV) individuals at the surface; once corrected for availability bias, this estimate indicates a summer presence of >12,700 devil rays in the study area. Rays were mostly observed alone even if occasionally, larger aggregations up to a maximum of 18 individuals were observed. Although observed throughout the study area, spatial modelling identified their preferred habitat to be over a broad strip connecting the Tuscan Archipelago to Eastern Sardinia, over a wide range of water depths ranging from 10 to 2000m. The observed seasonal changes in giant devil ray distribution in this study, combined with similar evidence from other areas in the Mediterranean, support the hypothesis that the species undertakes latitudinal migrations across the region, taking advantage of highly productive waters in the north during summer, and warmer southern waters during winter.

## Introduction

The giant devil ray *Mobula mobular* is one of the largest elasmobranchs, with a maximum disc width greater than 4.5m and a weight that can exceed 1.5 tonnes [1]. While the low reproductive potential common to all mobulids makes these rays vulnerable to fishing [2, 3], this may be particularly true for *M*. *mobular* with its limited range (the Mediterranean, possibly extending into the Northeast Atlantic [4, 5]). Until recently, no major fisheries specifically targeting giant devil rays were known to occur in the Mediterranean. Only small (3–4 animals annually) catches were known to be taken in a few specific locations (e.g. Algeria [6] and Sicily [7]). However, this picture substantially changed after the discovery of a directed seasonal fishery in the Levantine Sea off Gaza. Between January and April from 2005 to 2014, an annual mean catch of 83.2 rays was taken using purse seines, with peaks of 363 and 370 rays, respectively, in 2006 and 2013 [8]. Fishery bycatch further adds to giant devil ray mortality in the Mediterranean, in particular in the now illegal (since 2002) large-scale pelagic driftnets [8, 9, 10, 11, 12, 13], and to a smaller extent in bottom-set nets [14, 15, 16], purse seines for small pelagics [2], bottom trawls [15], pelagic trawls [17], longlines [16, 18, 19, 20], fixed traditional tuna traps [21, 22, 23]. In addition, there is evidence of sport fishing most often followed by release (e.g., youtube.com). Today, however, bycatch appears to be a lesser concern than directed catches off Gaza, particularly as a consequence of the progressive phasing out of pelagic driftnets in the Mediterranean, triggered by the 2002 ban. All sources of mortality considered, the conservation status of the giant devil ray was assessed as “Endangered, A4d” in IUCN’s Red List [24]. The species is also explicitly protected by Croatia and Malta, as well as being listed in Annex II (“List of endangered and threatened species”) to the Barcelona Convention Protocol on Specially Protected Areas and Biological Diversity in the Mediterranean (SPA/BD), in Appendix II (“Strictly protected fauna species”) to the Bern Convention on the Conservation of European Wildlife and Natural Habitats, and in both Appendices I and II to the Convention on the Conservation Migratory Species of Wild Animals.

Notwithstanding their status and the existing international legal framework for their protection, there are no enforced management measures in the Mediterranean or dedicated monitoring programmes [25]. Two scientific issues may be hampering proper management and conservation actions for this endangered elasmobranch: (1) uncertainty over its range caused by taxonomic difficulties; and (2) the intrinsic complexities of undertaking quantitative investigations of the population ecology and status of free-ranging elasmobranchs.

With respect to taxonomy, *M*. *mobular* can be confused with the spinetail devil ray *M*. *japanica*, a circumtropical species which is found in the tropical and warm-temperate North Atlantic, although not the Mediterranean [4]. Since *M*. *mobular* and *M*. *japanica* differ mainly in size (the latter being smaller) and in a number of subtle morphological and morphometric characters that cannot be detected during sightings at sea, it is possible that past reports of giant devil rays from the Atlantic—e.g., in coastal African waters from Morocco to Senegal, the Canary Islands, Madeira, the Azores, Portugal, and southern Ireland [1]—might have been in fact spinetail devil rays [4, 26]. Given that the only recorded mobulid in the Mediterranean is *M*. *mobular*, this uncertainty really only concerns its presence (or not) in Atlantic waters. Furthermore, the possibility that *M*. *mobular* is endemic to the Mediterranean should add to the need to develop effective conservation actions and monitoring.

Investigating the abundance and distribution of pelagic elasmobranch populations is challenging, due to their range, somewhat inaccessible habitat and the fact that, unlike mammals and reptiles, they do not have to come to the surface to breathe [27, 28]. Traditionally therefore, most of the existing knowledge of their biology and demographics has been obtained from fisheries data (e.g., [26]), which is problematic for species such as giant devil rays that are only subject to direct fishing on a small scale. Recent technical advances, such as the use of telemetry, have provided important new insights into the lives of larger elasmobranchs such as basking sharks, *Cetorhinus maximus* (e.g., [29]) and whale sharks, *Rhincodon typus* (e.g., [30]). Telemetry data have revealed previously unsuspected details about the extent and timing of horizontal and vertical movements of mobulids such as *M*. *japanica* [31], *M*. *tarapacana* [32], and *Manta alfredi* [33]. Of most relevance to this paper are the results of a telemetry experiment describing the movements and diving habits of *M*. *mobular* tagged in the Strait of Messina [34]. Telemetry data are invaluable in interpreting the results of aerial survey data as discussed later in this paper.

A separate development has been the increased use of aerial surveys to investigate the distribution, density and abundance of marine species such as cetaceans over large and remote areas, using the well-established approach of ‘Distance sampling’ e.g. [35]. They have been used in studies of large elasmobranchs such as manta rays [27], whale sharks [36, 37, 38], basking sharks [39], and great white sharks, *Carcharodon carcharias* [40]. Most recently, aerial surveys of the Adriatic Sea focussing primarily on cetaceans have generated valuable data on *M*. *mobular* in that Mediterranean sub-region, including the provision of novel information on giant devil ray distribution, density, and absolute abundance [25].

**Fig 1:**
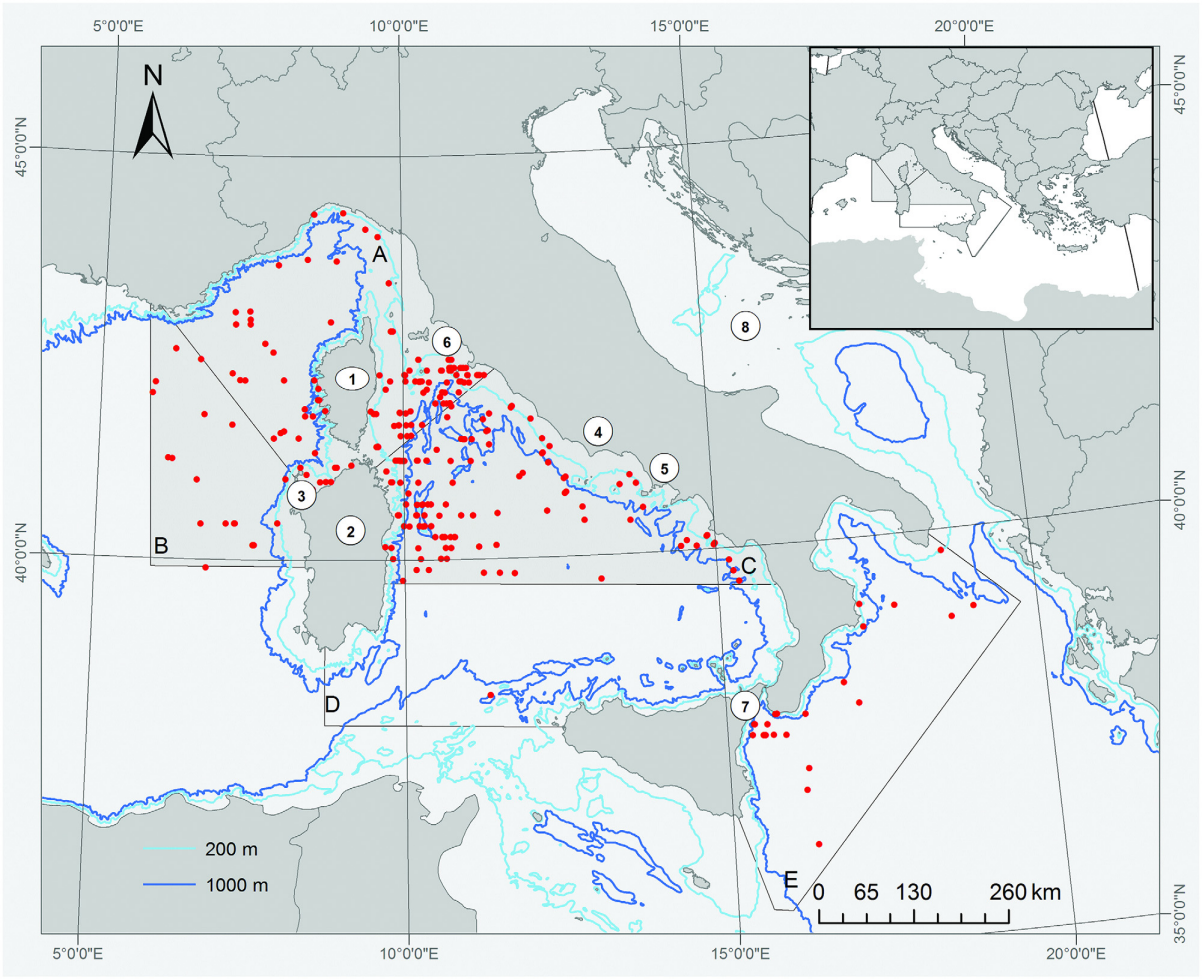
The study area subdivided into 5 sub-areas: (A) Pelagos Sanctuary; (B) Sardinian Sea; (C) Central Tyrrhenian Sea; (D) Southern Tyrrhenian Sea; and (E) Ionian Sea. Red dots are the locations of giant devilray sightings. The areas for which abundance estimates were obtained (A, B and C) are highlighted in grey. Numbers correspond to the following locations cited in the text: 1 –Corsica; 2 –Sardinia; 3 –Gulf of Asinara; 4 –Latium; 5 –Campania; 6 –Tuscan Archipelago; 7 –Strait of Messina; 8 –Adriatic Sea.

The present paper presents results on giant devil rays obtained from a series of aerial surveys, as in the case of [25], primarily aimed at investigating cetacean ecology, providing new insights into giant devil ray distribution, densities, and seasonality. Surveys were conducted between 2009 and 2013 in the North-Western Mediterranean Sea (Fig 1) [41, 42], including the waters of the Pelagos Sanctuary for Mediterranean Marine Mammals (hereafter `Pelagos Sanctuary`), a marine protected area established in 1999 by a treaty amongst France, Italy and the Principality of Monaco [43]. The establishment of this sanctuary to protect cetaceans, by helping the phasing out of the use of driftnets [44], has extended umbrella protection to giant devil rays.

## Materials and Methods

The study area (626,228 km^2^) covered by the aerial surveys is shown in Fig 1. Overall, between January 2009 and January 2014, 88 days were spent surveying 5 different sub-areas, some twice (Table 1).

**Table 1:** Subareas (letters indicate corresponding sectors in Fig 1), listed in chronological order of survey timing, with subarea surfaces and transect lengths (on average over 90% of the planned transect length was covered). Rows with bold lettering correspond to summer surveys, which were used for density and spatial analyses.

Sub-areas	Year	Season	Period (days flown)	Total surface (km^2^)	Planned transect length (km)	Surveyed transect length (km)
Pelagos Sanctuary (A)	2009	Winter	11 Jan– 22 Feb (20)	88,267	8,852	8,013
**Pelagos Sanctuary (A)**	**2009**	**Summer**	**20 Jul– 3 Aug (12)**	**88,267**	**8,852**	**8,502**
Ionian Sea (E)	2010	Spring	28 Apr– 14 May (12)	97,326	6,574	5,999
**Pelagos Sanctuary (A), Sardinian Sea (B), Central Tyrrhenian Sea (C)**	**2010**	**Summer**	**16 Jun– 4 Jul (14)**	**236,272**	**15,871**	**15,229**
Southern Tyrrhenian Sea (D)	2010–2011	Autumn—Winter	21–27 Oct (5), 13–20 Jan (7)	111,147	7,408	5,723
**Central Tyrrhenian Sea**	**2013**	**Summer**	**1–8 Aug (8)**	**93,216**	**5,930**	**6,211**
Southern Tyrrhenian Sea (D)	2014	Winter	7–16 Jan (10)	111,147	7,408	6,141

Surveys covered areas including broad bathyal plains at depths between 2000 and 2500 m associated with narrow continental shelves (sub-areas B, C, D, E, and the western portion of A), as well as shallower areas with extended shelves (eastern portion of A).

The aircraft used for all surveys was a two-engine high-wing *Partenavia* P-68 equipped with bubble windows to allow direct observation of the trackline under the plane, flying at the altitude of 750 feet (229 m) at a ground speed of 100 knots (185 km^-1^). Observations were performed by three experienced observers, two seated in the rear searching for animals through the opposite lateral bubble windows, the third in the co-pilot seat recording the data on a semi-automatic logging system. The plane flew along equally-spaced (15 km apart except in winter and summer 2009 when it was 10km) parallel transects, designed to provide equal coverage probability, along a total length of 60,895 km. Data collected for each sighting included: GPS-derived position, date and time of sighting, declination angle when the sighted animal(s) was seen abeam, aggregation size, and observer name. Primary search effort (searching carried out in acceptable conditions, i.e., with Beaufort ≤3 and good visibility) and altitude were recorded directly onto a laptop through data-logging software connected to the GPS. Additional information that may affect sightability (e.g., sea state, glare, cloud cover) was entered at the beginning of each transect and/or whenever changes occurred along the trackline. The perpendicular distance of each sighting from the trackline was derived trigonometrically from the altitude of the aircraft at the time of sighting and the declination angle to the sighting, measured with a *Suunto* clinometer.

Based upon the hypothesis that the peak presence of rays in the surface waters occurred throughout the study area during summer (as inferred from the high summer density of giant devil rays at the surface in sub-area A, starkly contrasting with their absence during winter; see Table 2), density estimates and spatial analyses have been carried out only with data coming from surveys conducted during those months (sub-areas A, B, and C; shaded in Fig 1).

**Table 2:** Total sightings (on track and off track) in the different sub-areas (capital letters refer to sub-area locations in Fig 1), and mean aggregation sizes (CV = coefficient of variation; CI = confidence interval; n/a = not applicable).

Sub-areas	Year	Season	Giant Devil Ray Sightings	Mean aggregation size	%CV	95% CI
Pelagos Sanctuary (A)	2009	Winter	0	n/a		
Pelagos Sanctuary (A)	2009	Summer	76	1.04	3.1	1–1.1114
Ionian Sea (E)	2010	Spring	22	1.67	16.7	1.1784–2.3572
Sardinian Sea (B)	2010	Summer	49	1.61	16.8	1.1343–2.2883
Pelagos Sanctuary (A)	2010	Summer	62	1.29	8.8	1.0825–1.5381
Central Tyrrhenian Sea (C)	2010	Summer	28	2.26	18.9	1.5527–3.2854
Southern Tyrrhenian Sea (D)	2010–11	Autumn-Winter	1	n/a		
Central Tyrrhenian Sea (C)	2013	Summer	60	1.35	9.3	1.1215–1.6251
Southern Tyrrhenian Sea (D)	2014	Winter	0	n/a		

Two methods to estimate giant devil ray abundance were used: (1) a design-based method [35], based on a survey design that ensures equal coverage probability across the study area; and (2) a model-based method [45, 46], in which line-transect sampling is combined with spatial analysis. The former has been the standard method used to estimate density and abundance from systematically collected survey data. Such estimates therefore enable comparison with many previous surveys and other areas. The latter approach potentially increases precision and also allows the production of density maps which can be especially valuable for developing conservation- and management-oriented actions. A further advantage is that abundance can be estimated for any subarea within the study area [45].

### Design-based method

A standard line transect distance sampling approach was implemented to obtain abundance and density estimates [35, 47]; survey design and sampling strategy were obtained using the latest available version of the dedicated software Distance [48]. Specific details and a full description of the methods and data collection protocols can be found in [41, 42].

Estimates were obtained using both the Conventional Distance Sampling, CDS [35] and the Multi Covariate Distance Sampling, MCDS [49] analyses engines in the Distance Software. In CDS, the detection probability is modelled solely as a function of the perpendicular distance to sightings.

The abundance in each area is estimated as:

$$\hat{N}=A\frac{n}{2L\hat{\mu }}\hat{E}\left[s\right]$$ (1)

where *A* is the surface (in km^2^), *L* is the total effort (in km), *n* is the number of sightings, $\hat{\mu }$ is the estimate of the effective strip width (*esw*), and $\hat{E}[s]$ is the estimate of the average group size.

In MCDS, the probability to detect objects depends not only on the distance from the trackline, but also on additional covariates that are directly incorporated in the detection probability estimation (e.g., [49, 50, 51]). Provided appropriate covariate data are collected, MCDS can potentially provide more precise estimates than CDS and reduce heterogeneity e.g., when regional differences in relevant environmental variables may differentially affect detection probability and lead to biased estimates if ignored when modelling the detection probability [51]. In MCDS the abundance is obtained using the Horvitz-Thompson estimator as it follows:

$$\hat{N}=A\frac{n}{2L}\hat{E}\left[s\right]\sum_{i=1}^{n}\frac{1}{\hat{\mu }\left({Ζ}_{i}\right)}$$ (2)

where the symbols are as for (Eq 1) and *z*_*i*_ represents the covariates.

A global detection function *g(y)*, the probability of detecting an object, given that it is at distance *y* from the random line [35] was obtained by pooling all the summer data (2009, 2010, 2013), stratified by area. The use of a pooled detection function is justified by the consistency among the surveys in terms of observers, aircraft and data collection protocols. Two additional detection functions were obtained: Pelagos summer stratified by year; and Central Tyrrhenian summer stratified by year, to compare the density estimates across years for the different areas. The Sardinian Sea was only surveyed in 2010, so its detection function was obtained from the global detection function.

**Fig 2:**
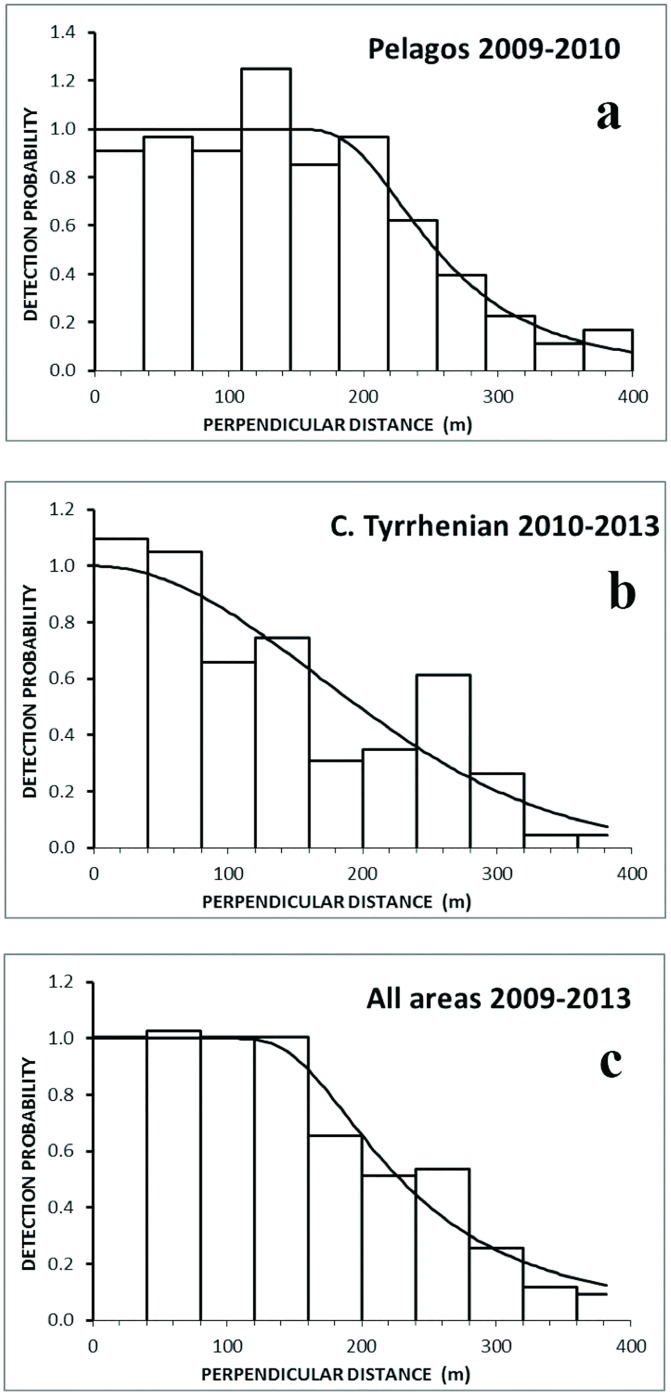
Perpendicular distance distribution (histograms), and fitted detection functions (lines): a) Pelagos Sanctuary, summer, stratified by year, b) Central Tyrrhenian Sea, summer, stratified by year, and c) all summer data pooled (2009, 2010 and 2013) and stratified by area.

**Fig 3:**
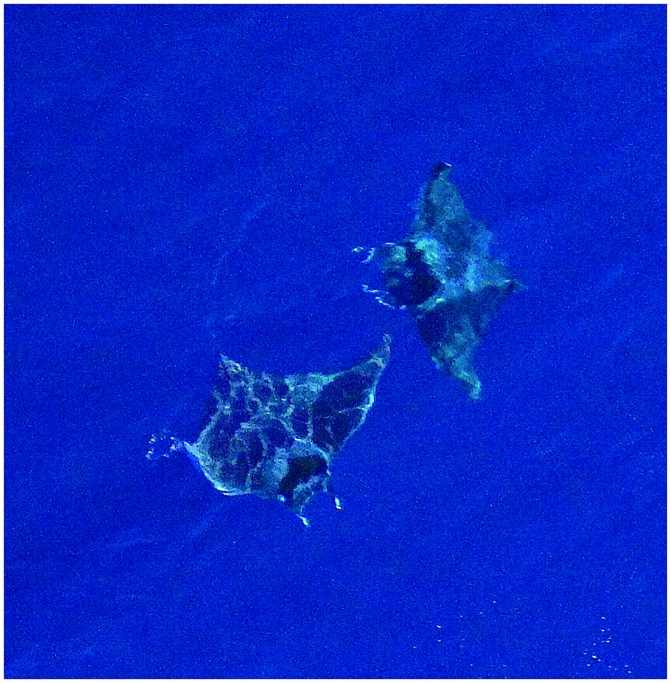
A pair of giant devil rays *Mobula mobular* photographed from the air. The general appearance of the body, the paired cephalic fins, the long tail and the dorsal colouration are diagnostic (photo by Elio Filidei, Jr.).

Perpendicular distance data were right truncated prior to the analysis after visual exploration of the frequency histograms of the observations plotted against the perpendicular distances themselves [35]. The truncation distance for all detection functions was set at 400m (Fig 2); this resulted in elimination of only one observation (at 564 m). A total of 266 observations remained: 21 for the Ionian Sea; 118 for the Central Tyrrhenian Sea; and 130 for Pelagos (Fig 3a, 3b and 3c). The additional explanatory covariates considered to model the detection probability in MCDS were: Beaufort, an assessment of overall sighting conditions and observer, all of them treated as factor variables. For both CDS and MCDS, the final selection of the model was based on the minimisation of the Akaike Information Criterion, AIC [35, 52], and examination of the performance of the qq-plot and the goodness of fit tests (chi-square, Kolmogorov-Smirnov and Cramer-von Mises).

### Model-based method

With this method, the perpendicular distance data are used to estimate a detection function, which allows abundance to be modelled as a function of physical and environmental data associated with the surveyed transects. Abundance then can be estimated for the entire study area through extrapolation and maps of created densities, using features of the environment to predict abundance, which can increase precision.

For the spatial analysis, a grid of cells was created covering sub-areas A, B and C, characterised according to potential spatial and environmental variables [53, 54]. A total of 26,944 cells were generated with a resolution of 17 km^2^. The covariates examined were:

1. spatial—latitude and longitude;
2. fixed—depth, distance to coast, distance to 200, 1000 and 2000m isobaths, slope, and aspect; and
3. dynamic: seasonal and annual means of sea surface temperature (SST), and chlorophyll concentration (Chl).

Depth was extracted from ETOPO [55], and its derivates were obtained using ArcGis 9. The SST and Chl-a values covered the period between 2009 and January 2014 and were obtained from SeaWiFS (between 2000 and 2010) and MODIS-Aqua sensors (from July 2002), and the SST of MODIS-Terra (from 2000) and MODIS-Aqua according to the operational lifespan of these three satellite platforms. Chl-a data from MODIS-Terra were not used due to the current low quality of the data. SST and Chl-a have wide coverage and both are available synchronously on a daily base (at the scale of the processes involved, i.e. within 12 h) at a medium resolution (geo-projected data at 4.6 km for MODIS and 9.2 km for SeaWiFS)[56].

All on-effort transects (Beaufort ≤3) were classified and divided into segments (mean = 3.1 km; max = 5.9 km) with homogeneous effort types, and under the assumption that little variability in the physical and environmental features occurred within each segment. These segments were subsequently associated to the attributes of the specific cell in which they fell, based on their spatial relationship.

Four models were created: (a) whole area for all years pooled together; (b) 2009 (only Pelagos); (c) 2010 (all areas); and (d) 2013 (only Central Tyrrhenian).

Using the count of animals in each segment as the response variable, the abundance of animals was modelled using a Generalized Additive Model (GAM) with a logarithmic link function and a Poisson error distribution. The general structure of the model was:

$$\hat{N_{i}}=\text{exp}\left[\text{ln}\left(a_{i}\right)+\theta_{0}+\sum_{k}f_{k}\left(z_{ik}\right)\right]$$ (3)

where the offset *a*_*i*_ is the effective search area for the *i* ^*th*^ segment (calculated as the length of the segment multiplied by twice the effective strip width, *esw*), *θ*_0_ is the intercept, *f*_*k*_ are smoothed functions of the explanatory covariates and *z*_*ik*_ is the value of the *k* ^*th*^ explanatory covariate in the *i* ^*th*^ segment. The *esw* was obtained from each of the detection functions, according to the spatial model to be created in terms of areas/years.

Abundance of animals was modelled directly, rather than using a two-step approach (modelling abundance of groups and modelling group sizes) given the low mean group size (see Table 2); almost 82% of the encounters (237) were of single animals, 9% of 2 animals (25), 8% from 3 to 8 animals (24) and 1% of 16 to 18 animals (2)). Therefore, the few encounters with more than one animal were treated as individual encounters with the same perpendicular distance.

Models were fitted using the R package ‘*mgcv*’ version 1.7–22 [57], and manually selected using three diagnostic indicators: (a) the Generalised Cross Validation score, GCV [58]; (b) the percentage of deviance explained; and (c) the probability that each variable was included in the model by chance. The decision to include/drop a term from the model was adopted following the criteria proposed in [59]. The point estimate of total abundance was obtained by summing the abundance estimates in all grid cells over the study area. Finally, to obtain the coefficient of variation and percentile-based 95% confidence intervals, using day as the resampling unit, 400 non-parametric bootstrap re-samples were applied to the whole modelling process. In each bootstrap replicate, the degree of smoothing of each model term was selected by the statistical package, thus incorporating some model selection uncertainty in the variance.

### Correcting for biases

In line transect surveys, two main biases—perception bias and availability bias—represent often substantive violations of a primary assumption of the method, i.e., that all animals on the trackline are detected [35], and both can cause an underestimation of abundance. Perception bias, when an observer misses an animal that is available to be seen, is customarily evaluated using an independent observer approach, i.e., with >1 observer independently searching the same area and recording data separately. This was not possible during the present surveys but the limitation of the surveys to good sighting conditions and the use only of experienced observers suggests that perception bias is likely to be small. By contrast, availability bias may be significant, especially for species such as the giant devil rays that unlike cetaceans do not need to come to the surface to breathe [27]. To account for the availability bias, we follow the approach of [25] for the giant devil rays in the Adriatic Sea. The authors of [25] derived a correction factor of 0.49 based upon telemetry data for three animals in the central Mediterranean between June and October 2007, which showed that devil rays spent some 49% of their daylight time (SD = 0.25) at a depth ≤10m, where they can be sighted under good conditions from the air [34]. Accordingly, corrected abundance estimates and CVs were obtained applying the following formula [60]:

$$CV\left(Nc\right)=\sqrt{CV{\left(Nc\right)}^{2}+CV{\left(\hat{a}\right)}^{2}}$$

Where $\hat{a}$ is 0.49 (from [25].

### Species identification

Due to their large size, distinctive body shape (including a very broad head with two prominent cephalic fins), a long, whip-like tail, and greyish dorsal pigmentation with a marked dark “collar” [4], giant devil rays can be unmistakeably identified from the air (Fig 3). Furthermore, *Mobula mobular* is the only member of the family Mobulidae found the Mediterranean Sea [4], which eliminates the problem of species misidentification.

### Ethical statement

No permit or approval was sought for this study, given that no takes were involved and the presence of observers is not considered to cause disturbance considering the fleeting and distant occurrence of the aircraft in the individual animals’ space.

## Results

The sightings data (n = 298) are summarised in Table 2. As noted earlier, almost 82% of the sightings were of single animals, with only a few sightings comprising larger numbers (up to 18). Rays were sighted in all sub-areas (Fig 1) and reported sightings were more frequent in the summer. The complete absence of sightings of devil rays during the 2009 winter survey over the Pelagos Sanctuary compares remarkably with the frequent sightings made in the same area using the same transects the following summer.

### Design-based approach

In examining the fits for the CDS and MCDS, no improvement was found for the MCDS and thus only CDS estimates were taken further. The best model for pooled data and for the Pelagos Sanctuary data was the hazard-rate function with no adjustment terms. For the Central Tyrrhenian Sea the best model was the half-normal with no adjustment terms. Fig 2 shows the perpendicular distance distribution (histograms), with fitted detection functions (lines) for the three models. The hazard rate function allows for a “shoulder” in the closer distances to the track line, indicating that the probability of detecting animals only decreases at certain distance from the track line (after around 200 m in the Pelagos Sanctuary and 80 m in the Tyrrhenian Sea). The abundance estimates are shown in Table 3.

**Table 3:** Density and uncorrected abundance estimates for the three sub-areas, obtained with design-based and model-based methods. *Esw*: effective strip width.

Sub-areas	Year	Sightings on track	Design-based method					Model-based abundance (%CV)
			*esw* (m) (%CV)	Density (indiv. km^-2^)	abundance	% CV	95% CI	
Pelagos Sanctuary	2009	68	268.1 (6.1%)	0.01558	1,375	22.9	882–2,144	1,346 (37.7)
Sardinian Sea	2010	18	248.2 (5.3%)	0.01700	931	27.1	540–1,606	978 (41.3)
Pelagos Sanctuary	2010	62	268.1 (6.1%)	0.02626	2,318	30.5	1,258–4,181	2,506 (27.1)
C. Tyrrhenian Sea	2010	58	206.5 (7.5%)	0.05191	4,838	26.6	2,890–8,101	4,031 (27.7)
C. Tyrrhenian Sea	2013	60	206.5 (7.48%)	0.03193	2,977	26.0	1,797–4,931	2,985 (48.2)
Pelagos Sanctuary	Total	130	248.2 (5.25%)	0.02145	1,893	19.0	1.308–2.740	1,849 (13.5)
Sardinian Sea		18		0.01700	931	27.1	540–1.606	978 (41.3)
C. Tyrrhenian Sea		118		0.03486	3,249	19.7	2.214–4.770	3,265 (15.1)
**Total**		**266**		**0.02571**	**6,074**	**13.4**	**4.673–7.894**	**6,092 (12.7)**

### Model-based approach

The best models for all years pooled together (all areas), for 2009 (Pelagos) and for 2010 (all areas) incorporated an interaction between the geographical covariates (Latitude and Longitude) and depth, while the model for 2013 (Central Tyrrhenian Sea) only incorporated Longitude and depth. In all cases covariates were highly significant. The total deviance explained was 14.8% for the global model, 30.5% for 2009, 24% for 2010, and 14.8% for 2013. The abundance estimates are shown in Table 3.

The results from the design-based and the model-based estimates correspond well, with an overall uncorrected abundance estimate for the study area (sum of sub-areas A, B and C) of just over 6, 000 (CV 13.4% for the design-based method and 12.7% for the model based method). Once corrected for availability bias, the total giant devil ray abundance estimate in the study area resulted to be around 12,500, with a CV of around 53% (12, 396, CV = 52.75% design-based; 12,722 CV = 52.57% model-based).

**Fig 4:**
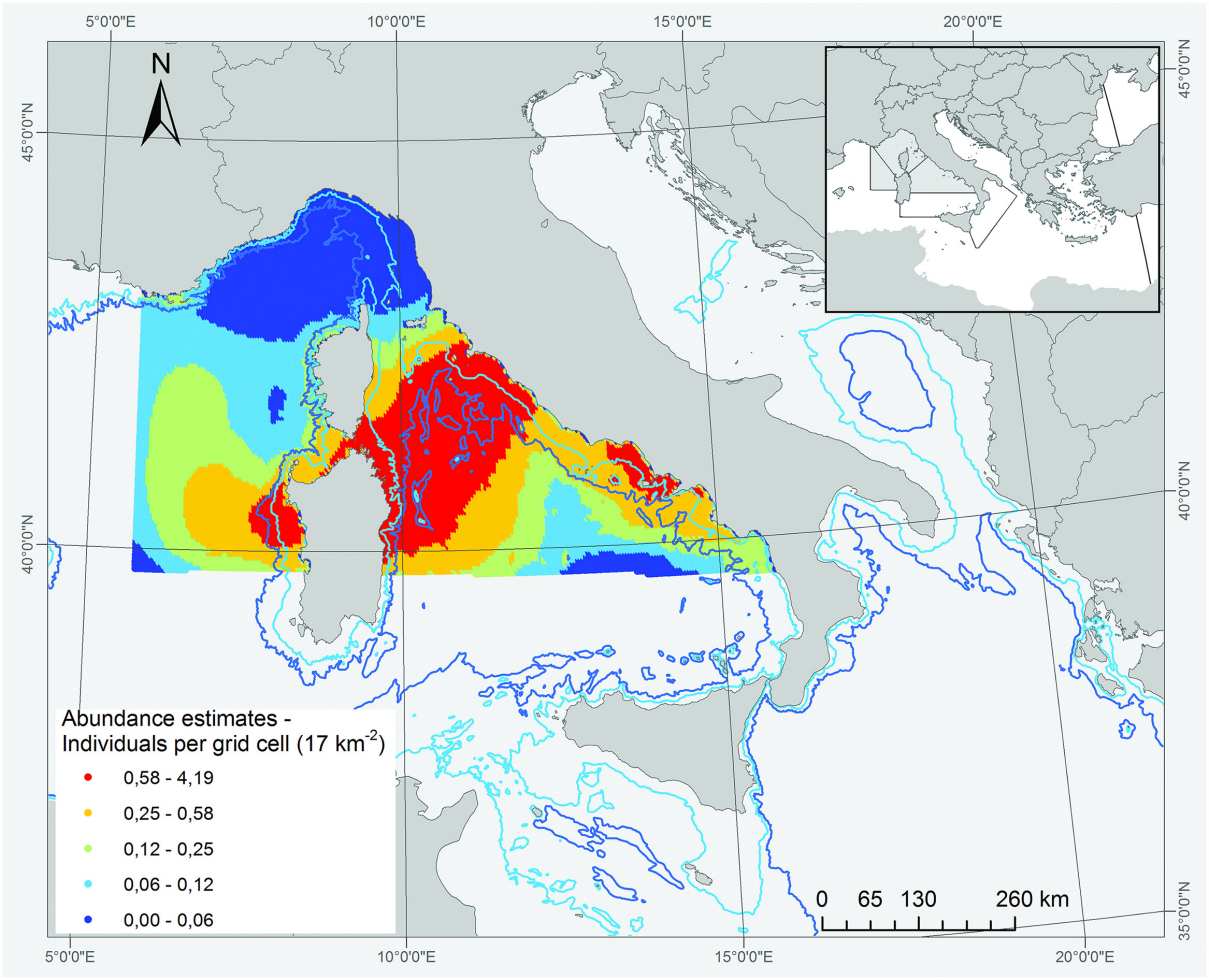
Predicted summer abundance of giant devil rays in the area considered for quantitative and spatial analysis, using geographic covariates (latitude and longitude) and depth as covariates.

The predicted summer abundance of giant devil rays for the area occupied by the Pelagos Sanctuary, the Sardinian Sea and the Central Tyrrhenian Sea (all years pooled) is shown in Fig 4. The model predicts the presence of areas of high abundance along a wide band connecting the coastline of central Italy with the northeastern portion of Sardinia, spilling slightly through the strait between Corsica and Sardinia into the Gulf of Asinara (northwest Sardinia). Smaller areas of high abundance are predicted off northwest Sardinia, extending to the west with decreasing density, as well as along the coast of southern Latium and Campania. Based on the model, the Pelagos Sanctuary is interested by areas of highest predicted devil ray abundance mostly in its southernmost part, i.e., along its border with the Central Tyrrhenian Sea and off northern Sardinia.

## Discussion

### Distribution and movements

In the present study, although sightings were scattered throughout the study area, a summer concentration of rays was apparent along a wide strip connecting the Tuscan Archipelago to Eastern Sardinia, over a wide range of water depths (Fig 1). Giant devil rays are believed to range widely throughout the Mediterranean Sea, both in neritic [2, 15, 16, 17] and offshore waters [34], over depths from few tens of metres to several thousand metres (giant devil rays are epipelagic batoids, which can dive up to at least 600–700 m [34]). However, in addition to the range given in the formal scientific literature, a search of other sources (e.g. images posted on the Internet by marine users), reveals these rays’ occurrence in many additional areas (e.g., Crete, Malta, Sicily Channel, Eastern Ionian Sea). This suggests that the species might be distributed more widely than previously thought, which is important when moving towards a full assessment of the species and evaluation of threats.

Strong seasonal changes in the presence of giant devil rays from specific Mediterranean locations suggest that they may undertake regular migrations. Migrations have been reported from several mobulid species [27, 31, 32]. Our data from the northern Pelagos Sanctuary show regular sightings in summer whereas despite extensive effort in good conditions, no animals were seen in winter (Table 2). The species is reported to aggregate in the Strait of Messina between late spring and summer [7], but is no longer seen there from around the middle of autumn [34]. In late autumn and winter, our data revealed low densities in the southern Tyrrhenian Sea (Table 2). Seasonal occurrence has also been reported for the Adriatic, where reports begin in April-May [16]. Observations of large aggregations of giant devil rays in winter in the southeastern corner of the Mediterranean, off the coast of Gaza [8], also suggests seasonal migrations. One hypothesis is that they migrate between the northern Mediterranean in summer and the southernmost shores in winter. Migratory behaviour of giant devil rays is consistent with these rays’ propensity for long-distance movements. The available tagging data for three animals showed movements from the Strait of Messina of 298 km (in 120 days), 337 km (in 120 days), and 278 km (in 60 days) [34]. Further tagging studies throughout the Mediterranean are required to address the question of giant devil ray migration, and how it might relate to seasonal changes in water circulation and primary productivity in the concerned areas.

Seasonal movements may be related to possible energetic advantages deriving from warmer waters if this species thermoregulates like other mobulids [61], or to the localized availability of high densities of prey [34] (e.g., mesopelagic and epipelagic fish in the Strait of Messina [7] and epipelagic fish in the central and southern Adriatic [16]).

During summer, the rays’ presence inside the Pelagos Sanctuary was higher along its southern boundaries, particularly in the Tyrrhenian Sea. This might be considered surprising given that the deep (down to 1,000m) and relatively productive [62] pelagic waters of the north-western Pelagos Sanctuary are a favoured habitat of one of giant devil rays’ prey species, *Meganyctiphanes norvegica* [63, 64], an euphausiid crustacean known to undergo diurnal vertical migrations in excess of 1 km [65]. One possible explanation might be that in the more northern Sanctuary waters, the giant devil rays spend more time at the greater depths frequented by euphausiids during the day, making them less available to sighting from the air.

Another driver of migratory behaviour in giant devil rays could be the species’ still unknown breeding habits [66]. Some evidence of sexual segregation is apparent from the catches (*n* = 30) of adults caught from 21 March to 10 April 2014 within the large aggregations observed off Gaza, 90% of which were males, some with sperm on the claspers [8]. By contrast, all the few (*n* = 5) specimens caught in the Strait of Messina between 1990 and 2003 were females [7].

### Density and abundance

The corrected absolute abundance estimate of >12,000 giant devil rays in the study area of the north western Mediterranean that covers approximately 10% of the Mediterranean is the first abundance estimate of the species in this region, and, despite the discussed uncertainties surrounding availability bias (see [25]), provides a possible basis for a future assessment of the conservation status and trends of *M*. *mobular* in a significant portion of its range.

Densities varied both in time (e.g., between successive years in the same area: 0.016 individuals km^-2^ in 2009 and 0.026 in 2010 in the Pelagos Sanctuary) and in space (consistently highest in the central Tyrrhenian Sea) (Table 3). The variation in time may reflect a real slight variation in density, or more likely, simply normal non-significant interannual variations due to environmental differences and/or the random effect of the sample size. Variation in space is reflected in the covariates retained in the model, depth being the only one with a clear ecological meaning. The geographic covariates (latitude and longitude) would be proxies for some unknown ecological factors. Both depth and other unknown factors most probably affect the distribution of prey, a major element affecting the spatial distribution of animals. To be able to detect a real trend in densities, a much lower CV would be needed during a longer term density estimates series. A power analysis is recommended to assess whether a real trend can be detected and under which circumstances. Mean density values calculated for the Adriatic Sea (0.018 indiv. km^-2^, 95% C.I.: 0.011–0.028, 22.6% CV) were slightly lower than in this study (mean around 0.25) but of the same order of magnitude [25].

Presudoreplication is unlikely to be an issue when pooling estimates from different regions and years. On the one hand, and stated above, movements of *M mobular* in this region appear to be very limited (around 3.3 km/day on average [34]), which reduces considerably the probability of duplicate sightings. On the other hand, as long as a duplicate sightings occur in different tracks (considered the independent sampling units), distance sampling theory shows that it does not result in bias; *var(n)* is estimated from variation in encounter rate between independent replicate lines [35]. Given the speed of the aircraft, it is not possible to have potential duplicates on the same sampling unit. Similarly, the fact that same animals may be present for example in Pelagos one survey year and in the Tyrrhenian in another sampling year again is not a problem as they are different sampling units.

## Conclusion

The present corrected abundance estimate of >12,000 is comparable (similar methods and time of year) with that for the Adriatic [25] of over 3,200 with similar large CVs (around 57%) which infers a total population size for the two areas of over 15,200. Despite the difficulties with respect to availability bias ([25]), this information provides for the possibility for the first time of undertaking a more quantitative assessment of the status of the giant devil rays in the Mediterranean. However, considerable uncertainties remain with respect to the biological parameters necessary for such an assessment (see discussion in [25]), as well as information on population structure, abundance throughout its range and levels of past and present removals (directed and bycaught). Information on the original population size e.g. from before the “driftnet era”–is unavailable [25].

Aerial surveys such as these, if carried out regularly, will provide the possibility for developing a comparable relative abundance series and thus an indication of trend and of the need for mitigation measures. However, to do this, further efforts (e.g. in telemetry and genetics, both for better estimates of time at the surface and an understanding of possible migrations and population structure) are required to reduce CV values and determine the proportion of the overall population being covered.

Without a better understanding of the levels of threats such as directed catches and bycatch, it is not possible at this stage to try to evaluate their likely effect on the status of the giant devil ray. Despite its formal protected status by a number of international and national regulations, there has been little systematic research into potential threats (including chemical pollutants and habitat degradation) and mitigation measures, or into monitoring trends in the population. If this protected status is to be meaningful, such information is essential.

## Supporting Information

S1 Dataset Giant devil ray sightings database. (XLSX)

## References

[1] Bigelow HB, Schroeder WC. Fishes of the Western North Atlantic. Part Two. Sawfishes, Guitarfishes, Skates and Rays. Chimaeroids. Memoir, Sears Foundation for Marine Research, Number I. New Haven; 1953.

[2] Notarbartolo di Sciara G, Serena F. Term embryo of *Mobula mobular* (Bonnaterre, 1788) from the northern Tyrrhenian Sea. Atti Soc Ital Sci Nat Mus Civ Stor Nat Milano 1988; 129:396–400.

[3] Dulvy NK, Pardo SA, Simpfendorfer CA, Carlson JK. Diagnosing the dangerous demography of manta rays using life history theory. PeerJ 2014; 2:e400 10.7717/peerj.400 24918029 PMC4045333

[4] Notarbartolo di Sciara G. A revisionary study of the genus *Mobula* Rafinesque, 1810 (Chondrichthyes, Mobulidae), with the description of a new species. Zool J Linn Soc. 1987; 91:1–91. 10.1111/j.1096-3642.1987.tb01723.x

[5] Cavanagh RD, Gibson C. Overview of the conservation status of cartilaginous fishes (Chondrichthyans) in the Mediterranean Sea. Switzerland and Malaga, Spain: IUCN, Gland; 2007.

[6] Hemida F, Mehezem S, Capapé C. Captures of the giant devil ray *Mobula mobular* Bonnaterre, 1788 (Chondrichthyes: Mobulidae) off the Algerian coast (southern Mediterranean). Acta Adriat. 2002; 43:69–76.

[7] Celona A. Catture ed avvistamenti di mòbula, *Mobula mobular* (Bonnaterre, 1788) nelle acque dello Stretto di Messina. Annales 2004; 14:11–18.

[8] Abudaya M. Assessment of the Gaza fishery of the giant devil ray (*Mobula mobular*). Final report to the Save our Seas Foundation; 2014.

[9] Northridge SP. Driftnet fisheries and their impacts on non-target species: a worldwide review. Rome. FAO Fisheries Technical Paper No. 320; 1991.

[10] Munoz-Chàpuli R, Notarbartolo di Sciara G, Séret B, Stehmann M. The status of the elasmobranch fisheries in Europe In: Earll RC, Fowler SL, editors. Proceedings of the 2^nd^ European Shark and Ray Workshop, London, Natural History Museum; 1994.

[11] Akyol O, Erdem M, Unal V, Ceyhan T. Investigations on drift-net fishery for swordfish (Xiphias gladius L.) in the Aegean Sea. Turk Vet Hayvanc Dergis 2005; 29:1225–1231.

[12] Walker P, Cavanagh RD, Ducrocq M, Fowler SL. Regional overviews: Northeast Atlantic (including Mediterranean and Black Sea). In: Sharks, Rays and Chimaeras: The Status of the Chondrichthyan Fishes (IUCN/SSC Shark Specialist Group); 2005. pp. 71–94.

[13] Banaru D, Dekeyser I, Imbert G, Laubier L. Non-target and released alive by-catch distributions observed during French driftnet fishery in the Northwestern Mediterranean Sea (2000–2003 database). J Ocean Res Data 2010; 3:33–45.

[14] Bini G (1967) Atlante dei pesci delle coste italiane Volume 1 Leptocardi—Ciclostomi—Selaci. Milano: Mondo Sommerso Ed; 1967.

[15] Bradai MN, Capapé C. Captures du diable de mer, *Mobula mobular*, dans le Golfe de Gabès (Tunisie Meridionale, Méditerranée Centrale). Cybium 2001; 25:389–391.

[16] Holcer D, Lazar B, Mackelworth P, Fortuna CM. Rare or just unknown? The occurrence of the giant devil ray (*Mobula mobular*) in the Adriatic Sea. J Appl Ichthyol. 2012; 29(1):134–144. 10.1111/jai.12034

[17] Scacco U, Consalvo I, Mostarda E. First documented catch of the giant devil ray *Mobula mobular* (Chondrichthyes: Mobulidae) in the Adriatic Sea. Mar Biodivers Rec. 2008; 2, e93 10.1017/S1755267209001110

[18] Marano G, Vaccarella R, Bello G, Pastorelli AM. Prime osservazioni sulla pesca di *Xiphias gladius* L. (Osteichthyes) nel Basso Adriatico. Thalassia Salentina 1983; 13:50–59.

[19] Bello G. The chondrichthyans of the Adriatic Sea. Acta Adriat. 1999; 40:65–76.

[20] Orsi Relini L, Cima C, Garibaldi F, Palandri G, Relini M, Torchia G. La pesca professionale con i palamiti galleggianti nel "Santuario dei Cetacei" del Mar Ligure: si tratta di attività ecocompatibili? Biol Mar Medit. 1999; 6:100–109.

[21] Boero F, Carli A. Catture di elasmobranchi nella tonnarella di Camogli (Genova) dal 1950 al 1974. Boll Mus Ist Biol Univ Genova 1979; 47:27–34.

[22] Silvi S. La manta di Favignana: eccezionale incontro in tonnara. Mondo Sommerso 1981; 251:56–61.

[23] Storai T, Zinzula L, Repetto S, Zuffa M, Morgan A, Mandelman J. Bycatch of large elasmobranchs in the traditional tuna traps (tonnare) of Sardinia from 1990 to 2009. Fish Res. 2011; 109:74–79. 10.1016/j.fishres.2011.01.018

[24] Notarbartolo di Sciara G, Serena F., Mancusi C. *Mobula mobular* In: IUCN Red List of Threatened Species. Version 2009.2. 2006. Available: http://www.iucnredlist.org/apps/redlist/details/39418/0

[25] Fortuna CM, Kell L, Holcer D, Canese S, Filidei E, Mackelworth P, Donovan G. Summer distribution and abundance of the giant devil ray (*Mobula mobular*) in the Adriatic Sea: baseline data for an iterative management framework. Sci Mar. 2014; 78(2):227–237. 10.3989/scimar.03920.30D

[26] Ebert DA, Stehmann MFW. Sharks, batoids, and chimaeras of the North Atlantic FAO Species Catalogue for Fishery Purposes. No. 7. Rome, FAO; 2013.

[27] Notarbartolo di Sciara G, Hillyer EV. Mobulid rays off eastern Venezuela (Chondrichthyes, Mobulidae). Copeia 1989; 3:607–615.

[28] Kessel ST, Gruber SH, Gledhill KS, Bond ME, Perkins RG. Aerial survey as a tool to estimate abundance and describe distribution of a carcharhinid species, the lemon shark, *Negaprion brevirostris* . J Mar Biol. 2013; 597383 10.1155/2013/597383

[29] Sims DW, Southall EJ, Richardson AJ, Reid PC, Metcalfe JD. Seasonal movements and behaviour of basking sharks from archival tagging: no evidence of winter hibernation. Mar Ecol Prog Ser. 2003; 248:187–196.

[30] Wilson SG, Polovina JJ, Stewart BS, Meekan MG. Movements of whale sharks (*Rhincodon typus*) tagged at Ningaloo Reef, Western Australia. Mar Biol. 2005; 148:1157–1166. 10.1007/s00227-005-0153-8

[31] Croll DA, Newton KM, Weng K, Galván-Magaña F, O’Sullivan J, Dewar H. Movement and habitat use by the spine-tail devil ray in the Eastern Pacific Ocean. Mar Ecol Prog Ser. 2012; 465:193–200. 10.3354/meps09900

[32] Thorrold SR, Afonso P, Fontes J, Braun CD, Santos RS, Skomal GB, Berumen ML. Extreme diving behaviour in devil rays links surface waters and the deep ocean. Nat Commun. 2014; 5:4274 10.1038/ncomms5274 24983949 PMC4102113

[33] Braun CD, Skomal GB, Thorrold SR, Berumen ML. Diving behavior of the reef manta ray links coral reef with adjacent pelagic habitat. PloS ONE 2014; 9(2): e88170 10.1371/journal.pone.0088170 24516605 PMC3916408

[34] Canese S, Cardinali A, Romeo T, Giusti M, Salvati E, Angiolillo M, Greco S. Diving behaviour of giant devil ray in the Mediterranean Sea. Endanger Species Res. 2011; 14:171–176. 10.3354/esr00349

[35] Buckland ST, Anderson DR, Burnham KP, Laake JL, Borchers DL., Thomas L. Introduction to distance sampling: estimating abundance of biological populations. Oxford: Oxford University Press; 2001.

[36] Burks CM, Driggers WB, Mullin KD. Abundance and distribution of whale sharks (*Rhincodon typus*) in the northern Gulf of Mexico. Fish. Bull. (Wash. D. C.) 2006; 104:579–584.

[37] Rowat D, Gore M, Meekan MG, Lawler IR, Bradshaw CJA. Aerial survey as a tool to estimate whale shark abundance trends. J Exp Mar Bio Ecol. 2009; 368:1–8. 10.1016/j.jembe.2008.09.001

[38] Hueter RE, Tyminski JP, de la Parra R. Horizontal movements, migration patterns, population structure of whale sharks in the Gulf of Mexico and Northwestern Caribbean Sea. PloS ONE 2013; 8(8): e71883 10.1371/journal.pone.0071883 23991000 PMC3749210

[39] Wilson SG. Basking sharks (*Cetorhinus maximus*) schooling in the southern Gulf of Maine. Fish. Ocean. 2004; 13(4):283–286.

[40] Dicken ML, Booth AJ. Surveys of white sharks (*Carcharodon carcharias*) off bathing beaches in Algoa Bay, South Africa. Mar Freshw Res. 2013; 64:530–539. 10.1071/MF12336

[41] Lauriano G, Pierantonio N, Donovan G, Panigada S. Abundance and distribution of *Tursiops truncatus* in the Western Mediterranean Sea: an assessment towards the Marine Strategy Framework Directive requirements. Mar Environ Res. 2014; 100:86–93. 10.1016/j.marenvres.2014.04.001 24784442

[42] Panigada S, Lauriano G, Burt L, Pierantonio N, Donovan G. Monitoring winter and summer abundance of cetaceans in the Pelagos Sanctuary (Northwestern Mediterranean Sea) through aerial surveys. PloS ONE 2011; 6(7):e22878 10.1371/journal.pone.0022878 21829544 PMC3146501

[43] Notarbartolo di Sciara G, Agardy T, Hyrenbach D, Scovazzi T, Van Klaveren P. The Pelagos Sanctuary for Mediterranean marine mammals. Aquat Conserv. 2008; 18:367–391. 10.1002/aqc.855

[44] Cornax MJ, Pastor X, Aguilar R, Cator J. "Thonaille": the use of driftnets by the French fleet in the Mediterranean. Oceana Report; 2007.

[45] Hedley SH, Buckland ST and Borchers DL. Spatial modelling from line transect data. J Cetacean Res Manag. 1999; 1(3): 255–264

[46] Marques FFC. Estimating wildlife distribution and abundance from line transect surveys conducted from platforms of opportunity. PhD Thesis. University of St Andrews, Scotland. 2001. Available: http://research-repository.st-andrews.ac.uk/bitstream/10023/3727/3/FernandaFCMarquesPhDThesis.pdf

[47] Thomas L, Laake JL, Rexstad E, Strindberg S, Marques FFC, Buckland ST, Borchers DL, Anderson DR, Burnham KP, Burt ML, Hedley SL, Pollard JH, Bishop JRB, Marques TA. Distance 6.0. Release “2” Research Unit for Wildlife Population Assessment, University of St. Andrews, UK 2009 Available: http://www.ruwpa.st-and.ac.uk/distance/

[48] Thomas L, Buckland ST, Rexstad EA, Laake JL, Strindberg S, Hedley SH, Bishop JRB, Marques T, Burnham KP. Distance software: design and analysis of distance sampling surveys for estimating population size. J Appl Ecol. 2010; 47: 5–14. 20383262 10.1111/j.1365-2664.2009.01737.x PMC2847204

[49] Marques FFC, Buckland ST. Covariate models for the detection function In: Buckland ST, Anderson DR, Burnham KP, Laake JL, Borchers DL, et al, editors. Advanced distance sampling: estimating abundance of biological populations. Oxford: Oxford University Press; 2004.

[50] Ramsey FL, Wildman V, Engbring J. Covariate adjustments to effective area in variable-area wildlife surveys. Biometrics 1987; 43(1):1–11.

[51] Marques FFC, Buckland ST. Incorporating covariates into standard line transect analyses. Biometrics 2003; 59(4):924–935. 14969471 10.1111/j.0006-341x.2003.00107.x

[52] Akaike H. A new look at the statistical model identification. IEEE Trans Automat Contr. 1974; 19(6):716–723. 10.1109/TAC.1974.1100705

[53] Cañadas A and Hammond PS. Model-based abundance estimate of bottlenose dolphins off Southern Spain: implications for conservation and management. J Cetacean Res Manag. 2006; 8(1):13–27

[54] Cañadas A and Hammond PS. Abundance and habitat preferences of the short-beaked common dolphin (*Delphinus delphis*) in the South-western Mediterranean: implications for conservation. Endanger Species Res. 2008; 4:309–331.

[55] ETOPO. Available: http://www.earthmodels.org/data-and-tools/topography/etopo

[56] Druon JN, Panigada S, David L, Gannier A, Mayol P, Arcangeli A, Cañadas A, Laran S, Di Méglio N, Gauffier P. Potential feeding habitat of fin whales in the western Mediterranean Sea: an environmental niche model. Mar Ecol Prog Ser. 2012; 464:289–306. 10.3354/meps09810

[57] Wood SN. R-Manual: the MGCV package. Version 1.3–22. Technical Report; 2006.

[58] Wood SN. Modelling and smoothing parameter estimation with multiple quadratic penalties. J Roy Statist Soc B 2000; 62(2):413–428

[59] Wood SN. MGCV: GAMs and generalized ridge regression for R. R News 2001; 1(2): 20–25.

[60] Heide-Jorgensen MP, Laidre KL, Hansen RG, Burt ML, Simon M, Borchers DL, Hansen J, Harding JK, Rasmussen M, Dietz R, Teilmann J. Rate of increase and current abundance of humpback whales in West Greenland. J Cetacean Res Manag. 2012; 12(1):1–14.

[61] Alexander RL. Evidence of brain-warming in the mobulid rays, *Mobula tarapacana* and *Manta birostris* (Chondrichthyes: Elasmobranchii: Batoidea: Myliobatiformes). Zool J Linn Soc. 1996; 118:151–164. 10.1111/j.1096-3642.1996.tb00224.x

[62] Jacques G. L'oligotrophie du milieu pélagique de Méditerranée occidentale: un paradigme qui s'éstompe? Bull Soc Zool France 1989; 114(3):17–29.

[63] Casanova B. Répartition bathymétrique des euphausiacés dans le bassin occidental de la Méditerranée. Rev Trav Inst Peches Marit. 1970; 34(2):205–219.

[64] Orsi Relini L, Cappello M. The fin whale and other pelagic filterers as samplers of *Meganyctiphanes norvegica* . Rapp Comm Int Mer Médit. 1992; 33:263.

[65] Mauchline J. The biology of mysids and euphausiids. Adv Mar Biol. 1980; 18:1–681.

[66] Couturier LIE, Marshall AD, Jaine FRA, Kashiwagi T, Pierce SJ, Townsend KA, Weeks SJ, Bennett MB, Richardson AJ. Biology, ecology and conservation of the Mobulidae. J Fish Biol. 2012; 80:1075–1119. 10.1111/j.1095-8649.2012.03264.x 22497374

